# The Complete Chloroplast Genome of *Camellia tianeensis* (*Camellia* L.) and Phylogenetic Relationships with Other Plants of the Genus *Camellia*

**DOI:** 10.3390/genes16101217

**Published:** 2025-10-15

**Authors:** Juyan Chen, He Li, Lunxiu Deng

**Affiliations:** Guizhou Academy of Forestry, Guiyang 550005, China; jychen2008@163.com (J.C.);

**Keywords:** *Camellia tianeensis*, sect. *Chrysantha*, chloroplast genome, phylogenetics, comparative genomics

## Abstract

Background/Objectives: Species within section *Chrysantha* represent the only camellias known to produce golden-yellow petals. The primary objectives of this study were to characterize the chloroplast genome structure of *Camellia tianeensis* and to elucidate its phylogenetic position with sect. *Chrysantha*. Methods: The complete chloroplast genome of *C. tianeensis* was sequenced, assembled, and annotated. Phylogenetic inference was conducted using maximum likelihood and Bayesian methods based on complete chloroplast genomic sequences. Results: The chloroplast genome of *C. tianeensis* is 156,865 bp in length and exhibits a typical quadripartite structure consisting of a large single-copy (LSC) region (86,579 bp), a small single-copy (SSC) region (18,236 bp), and two inverted repeat (IR) regions (26,025 bp each). The genome encodes 164 genes, including 111 protein-coding genes, 45 tRNAs, and 8 rRNA genes. The overall GC content was 37.32%, with regional values of 35.33% (LSC), 30.59% (SSC), and 42.99% (IRs). Sixty-nine simple sequence repeats (SSRs) were identified, predominantly mononucleotide repeats, Thirty-eight dispersed repeats were categorized into three types (forward, reverse, and palindromic), with no complement repeats detected. Phylogenetic analysis strongly supported that *C. tianeensis* is a member within sect. *Chrysantha*. Conclusions: *C. tianeensis is* phylogenetically closely related to *C. huana*, forming a well-supported clade. This study enhances the molecular research available for sect. *Chrysantha* and provides a genomic foundation for future phylogenetic and taxonomic studies in this group.

## 1. Introduction

Plants of section *Chrysantha* Chang (genus *Camellia*, family Theaceae) are characterized by their “pure yellow flowers like gold” and represent the only camellia with golden-yellow petals. Their round and delicate buds have earned them the titles “Queen of the Tea Family” and the “Giant Panda of the Plant World” [1]. Currently, 42 species and 5 varieties within sect. *Chrysantha* are recognized, native to southern China and Vietnam, with the majority distributed in Guangxi Province and a few in Guizhou, Yunnan, and Sichuan Provinces [2]. Sect. *Chrysantha* comprises a group of subtropical plants that thrive in warm and humid climates. These plants are efficient in nutrient utilization and exhibit strong resistance to waterlogging. They have low soil requirements and can grow in slightly acidic to neutral soils [3]. Sect. *Chrysantha* species are evergreen shrubs or small trees with yellow-brown, nearly smooth bark. Their leaves are leathery, oblong, lanceolate, or rarely oblanceolate, with dark-green upper surfaces, inconspicuous reticulate veins, and serrulate margins. The axillary flowers are yellow and solitary, with 10–13 fleshy, glabrous petals; the outer whorl is suborbicular, while the inner whorl is obovate or elliptic. The superior ovary is 3-loculed and glabrous, with 3–4 glabrous styles. Flowering occurs from December to March. The capsule is depressed-globose, measuring 3.5 cm length and 4.5 cm in width, containing 2–3 seeds per locule with a concave apex. The seed are brown, glossy, hemispherical, and 1.5–2 cm in diameter [4]. Plants in this section possess significant ornamental, medicinal, and economic value [5,6,7,8].

*C. tianeensis* S. Yun Liang et Y. T. Luo is a member of sect. *Chrysantha*. First described as a new species by Liang Shengye et al. in 1995 [9], it is characterized by elliptic leaves with 6–7 pairs of lateral veins, solitary flowers that are purplish-red or light-red in bud and turn yellowish after opening, and brown seeds. However, Flora of China treated it as a synonym of *Camellia huana* [10]. To resolve this taxonomic discrepancy, we conducted an examination of specimens, field observations of wild populations, micro-morphological analyses of leaves and pollen, and multi-year introduction and cultivation studies, ultimately confirming *C. tianeensis* as a distinct species and identifying *C. liberofilamenta* as a synonym of *C. huana* [11]. Although studies on its distribution, conservation, cultivation, and population characteristics have been conducted [12,13,14,15,16,17], molecular data for phylogenetic placement were previously lacking. This study reports the complete chloroplast genome of *C. tianeensis*, providing genetic information that will support further classification, evolutionary studies, and utilization of this species.

## 2. Materials and Methods

### 2.1. Material Collection, DNA Extraction, and Sequencing

**Figure 1:**
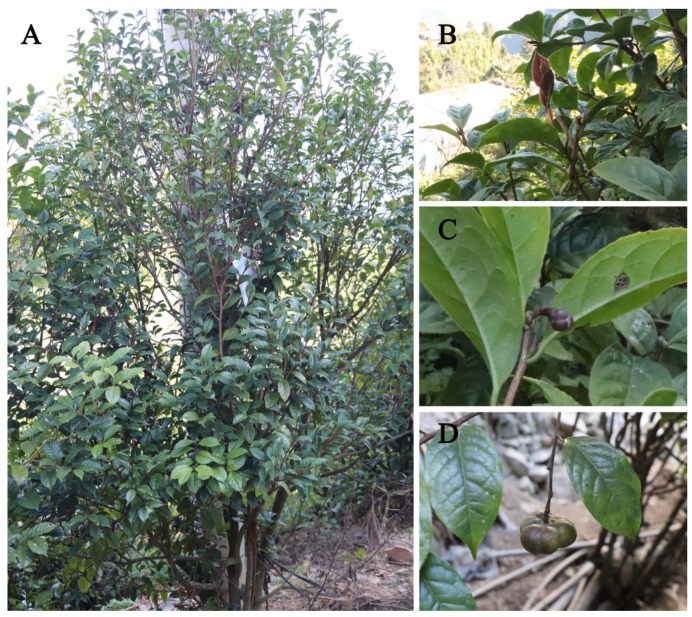
Morphology of *C. tianeensis*. ((**A**): habitat; (**B**): branch; (**C**): bud; (**D**): fruit).

Samples of *C. tianeensis* were collected from the Forestry Bureau of Ceheng County, Guizhou Province, China (N 24.98465303°, E 105.81570840°; Figure 1). Voucher specimens were deposited in the Tree Specimen Laboratory of the Guizhou Academy of Forestry (GZAF, accession no. LH-20221101). Fresh young leaves were collected, and chloroplast DNA was extracted using an optimized CTAB method [18]. DNA integrity was assessed by 1% agarose gel electrophoresis, and purity and concentration were determined using a spectrophotometer. Sequencing libraries were constructed through fragmentation, end repair, and adapter ligation, and high-throughput sequencing was performed on the NovaSeq 6000 platform.

### 2.2. Assembly and Annotation of the Chloroplast Genome

Clean reads were assembled de novo using GetOrganelle 1.7.5.3 [19] to obtain a circular chloroplast genome. Annotation was performed using CPGAVAS2 [20] with BLAST 2.17.0 comparison and manual correction. The annotated sequence was submitted to NCBI (GenBank ID: PP187689). A genome map was generated using OGDRAW [21].

### 2.3. Repeat Sequence Analysis and Codon Preference

SSRs were identified using MISA 2.1 [22] with the following thresholds: ≥10 repeats for mononucleotides, ≥5 for dinucleotides, ≥4 for trinucleotides, and ≥3 for tetra-, penta-, and hexanucleotides [23]. Dispersed repeats were detected using reputer [24] with a Hamming distance of 3 and a minimum repeat size of 30 bp. Repeat types included forward (F), reverse (R), complement (C), and palindromic (P). Codon usage and relative synonymous codon usage (RSCU) were analyzed with CodonW 1.4.2 [25] and visualized using R v4.0.5.

## 3. Phylogenetic Analysis

Complete chloroplast genome sequences of 22 sect. *Chrysantha* species were downloaded from NCBI. *Camellia pyxidiacea* (GenBank ID: OP058659) was used as the outgroup. Sequences were aligned with MAFFT7 v7 [26], and maximum likelihood (ML) phylogeny was inferred using MEGA X [27] under the GTR+I+G model. Support values were calculated with 1000 bootstrap replicates in IQ-TREE v2.2.0 [28]. The optimal model (HKY+G+I) was identified using MrModeltest v2.3, and a Bayesian inference (BI) was constructed using MrBayes v3.2.7 [29] under the HKY+G+I model selected by MrModeltest v2.3. Trees were visualized using iTOL v4 [30].

## 4. Results

**Figure 2:**
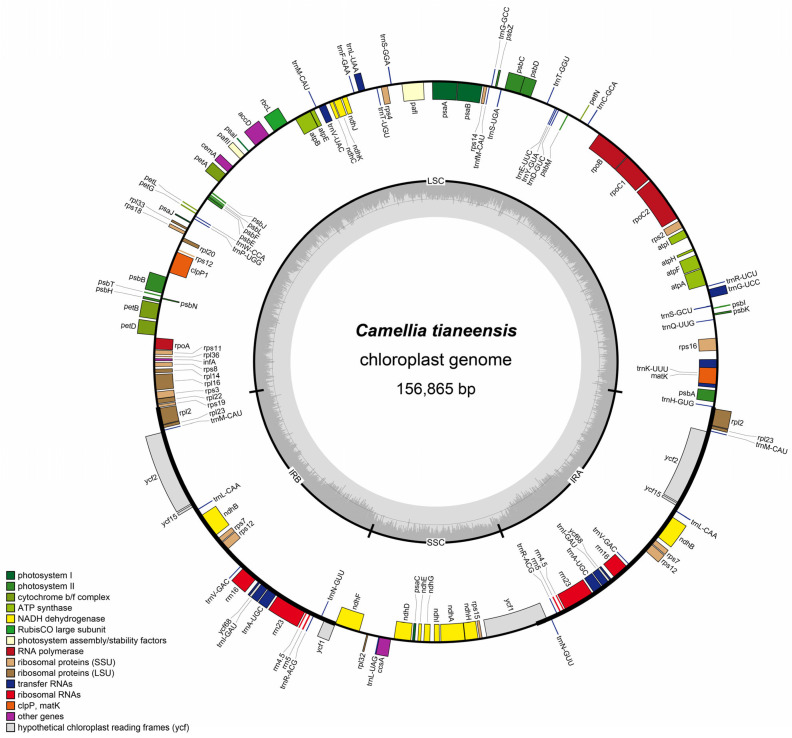
Circular gene map of chloroplast genomes of *C. tianeensis*. Genes on the outside and inside of the circle are transcribed in clockwise and counterclockwise directions, respectively.

**Figure 3:**
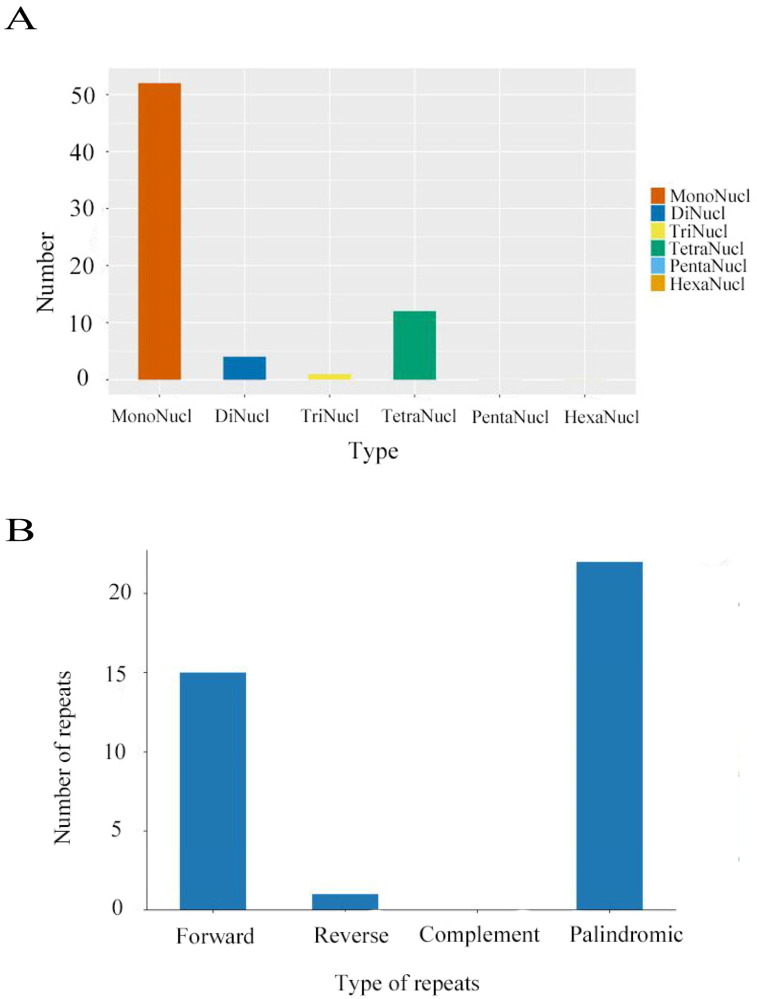
Analyses of repeated sequences. (**A**): The numbers of the six SSR types. (**B**): The numbers of the four long repeat types.

**Figure 4:**
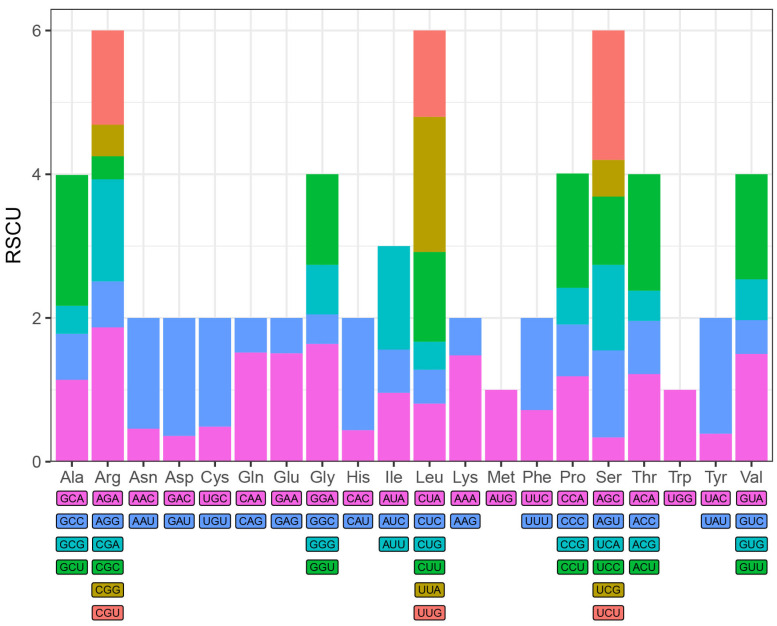
RSCU analysis of each amino acid. The histogram presents each amino acid codon usage within *C. tianeensis* cp genome, and the color of the histogram corresponds to the color of codons.

This study successfully assembled the complete chloroplast genome of *C. tianeensis* (Figure 2) which has a total length of 156,865 bp and exhibits a typical quadripartite structure consisting of one large single-copy (LSC) region (86,579 bp), one small single-copy (SSC) region (18,236 bp), and two inverted repeat (IR) regions (26,025 bp each). The overall GC content was 37.32%, with regional distributions of 35.33% in the LSC, 30.59% in the SSC, and 42.99% in the IRs. Genome annotation identified a total of 164 genes, including 111 protein-coding genes, 45 tRNAs genes, and 8 rRNAs genes. A total of sixty-nine simple sequence repeats (SSRs) were detected throughout the chloroplast genome, comprising 52 mononucleotide repeats (21 A and 31 T), 4 dinucleotide repeats (3 AT and 1 TA), 1 trinucleotide repeat (TTC), and 12 tetranucleotide repeats. Mononucleotides repeats were the most abundant type, significantly outnumbering other repeat categories (Figure 3A). Additionally, thirty-eight dispersed repeats were identified and classified into three types: 15 forward (F) repeats, 1 reverse (R) repeat, and 22 palindromic (P) repeats. No complement (C) repeats were observed (Figure 3B). Codon usage analysis identified 61 codons encoding 20 amino acids in addition to the three stop codons (UAA, UAG, UGA). Among the 27,091 codons identified, leucine (Leu) was the most frequent (2819 codons, 10.40%), while cysteine (Cys) was the least (296 codons, 1.09%), excluding the stop codons. Thirty-one codons had a relative synonymous codon usage (RSCU) value greater than 1. Of these, 13 ended with A, 16 with U, and 1 with G (UUG). The predominance of A/U-ending codons indicates a clear A/U bias in the codon usage of the *C. tianeensis* chloroplast genome (Figure 4).

**Figure 5:**
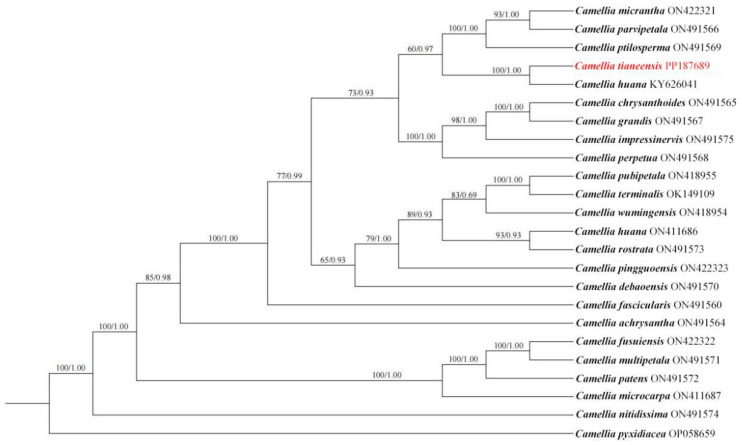
Phylogenetic tree constructed using the maximum likelihood (ML) and Bayesian inference (BI) methods based on the complete chloroplast genome sequences. Numbers above branches indicate ML bootstrap values (BS, **left**) and the posterior probabilities (PP, **right**).

A phylogenetic tree reconstructed from 22 published complete chloroplast genomes of species from section *Chrysantha* confirmed that *C. tianeensis* is a member of this section (Figure 5). It formed a well-supported clade with *C. liberofilamenta* (BS/PP = 100/1.00). Most nodes of the tree were highly supported, and *C. pyxidiacea*, used as the outgroup, was clearly separated from the clade containing sect. *Chrysantha.*

## 5. Discussion

Chloroplasts, the primary organelles responsible for photosynthesis, possess independent and complete genomes and exhibit uniparental inheritance in most plant species [31]. Owing to its conservative nature and sequence variability, the chloroplast genome has been extensively utilized in various research fields, including plant taxonomic revision, population genetics, genetic diversity, phylogenetic analysis, and historical population dynamics [31,32]. Since the advent of whole chloroplast genome sequencing, it has attracted widespread scholarly interest and has been applied to address numerous important botanical questions, such as resolving ambiguous taxonomic classifications. By analyzing data from the NCBI database, we obtained chloroplast genomic information for over 100 species within the genus *Camellia*. These chloroplast genomes exhibit relatively limited size variation, ranging from 150 to 160 kb, and all share a typical quadripartite structure with highly conserved structural features. The level of sequence conservation in chloroplast DNA is positively correlated with GC content. In this study, we contributed to the enrichment of *Camellia* chloroplast genomic resources by submitting the complete chloroplast genome sequence of *C. tianeensis* to the NCBI database.

Simple sequence repeats (SSRs), which consist of 1–6 nucleotide tandem repeats, are widely used in plant species identification, genetic mapping, population genetics, systematic evolution, and studies of genetic diversity in germplasm resources due to their abundance and high polymorphism [33,34,35]. In this study, we identified 69 SSRs in the chloroplast genome of *C. tianeensis*, comprising 52 mononucleotide, 4 dinucleotide, 1 trinucleotide (TTC-1), and 12 tetranucleotide repeats. Mononucleotide SSRs were the most abundant, outnumbering other types significantly. Additionally, 38 dispersed repeats were detected, with no complementary (C) repeats observed. The sequence composition of these SSR loci is consistent with previous reports, confirming that polyA and polyT repeats dominate [36]. The SSR loci identified here will provide a foundation for further molecular genetic analyses of sect. *Chrysantha*.

Codon usage bias refers to the non-uniform utilization of synonymous codons that encode the same amino acid within an organism. Over the course of evolution, certain codons become preferentially used, forming a set of optimal codons. This preference is influenced by factors such as mutation, selection, gene length, gene function, and genetic drift. Since codon usage patterns vary across species, they can serve as an indicator of phylogenetic relatedness [37]. The Relative Synonymous Codon Usage (RSCU) is defined as the ratio of the observed frequency of a codon to its expected frequency under equal usage. An RSCU value less than 1 indicates that the codon is used less frequently than other synonymous codons; a value greater than 1 suggests higher relative usage; and an RSCU equal to 1 implies no preferential usage [38]. Among amino acids, only tryptophan (Trp) and methionine (Met) exhibit an RSCU value of 1, as each is encoded by a single codon; hence, no codon bias exists for these residues. In *C. tianeensis,* codons show a preference for those ending in A/U, which is consistent with findings from Zhang Xiaoyu’s study on plants in the sect. *Chrysantha* [4].

Phylogenetic analysis serves as a powerful tool for elucidating affinities among species. In their study, Wei et al. [39]. reconstructed the phylogenetic relationships of yellow-flowered *Camellias* using multiple molecular datasets and proposed a revised taxonomic treatment for sect. *Chrysantha*, recognizing 20 species within this section. Although their work provided a comprehensive analysis of the section, it did not include *C. tianeensis*, which was then regarded as a synonym of *C. huana*. Furthermore, the chloroplast genome data used by Wei et al. [39] were incomplete, comprising only the small single-copy (SSC) region rather than the full chloroplast genome. Previous phylogenetic reconstructions based on single-copy homologous genes suggested that golden camellia species in closer geographic proximity exhibit stronger phylogenetic relationships [40]. In the present study, however, phylogenetic analysis using complete chloroplast genome sequences strongly supports a sister relationship between *C. tianeensis* and *C. huana*. This finding offers important insights into the evolutionary history of sect. *Chrysantha* at the genomic level. Additionally, combined evidence from this study and our earlier work [11] suggests that the published chloroplast genome sequence of *C. huana* (accession no. ON411686) may have been misidentified. The molecular reassessment of *C. tianeensis* presented here not only clarifies its phylogenetic placement but also provides a foundation for future research on molecular systematics and population evolution within this section.

## 6. Conclusions

In this study, the chloroplast genome of *C. tianeensis* was sequenced and characterized for the first time using high-throughput sequencing technology. The repeat sequences and codon usage bias were systematically analyzed. The chloroplast genome was found to be 156,865 bp in length and contained 164 genes. Four types of simple sequence repeats (SSRs) were identified among the repeat sequences, although no complement (C) repeats were detected. Analysis of codon usage preference across the chloroplast genome revealed a tendency for codons to end with A/U. Phylogenetic reconstruction based on the chloroplast genome placed *C. tianeensis* within the same clade as other species in section *Chrysantha*. These genomic data provide valuable insights for elucidating phylogenetic relationships within section *Chrysantha*. Furthermore, this study offers a theoretical foundation and technical support for the rational utilization and effective conservation of this species in the future.

## Data Availability

The genome data that supported the findings of this study are openly available in GenBank of NCBI at [https://www.ncbi.nlm.nih.gov (accessed on 1 October 2025)] under accession no. PP187689.

## Abbreviations

The following abbreviations are used in this manuscript:

LSC	large single-copy region
SSC	small single-copy region
IR	inverted-repeat regions
SSR	simple sequence repeat
RSCU	relative synonymous codon usage
F	Forward
R	Reverse
C	Complement
P	Palindromic
